# Supplementary material for: Melt focusing along lithosphere–asthenosphere boundary below Axial volcano
Source: Nature. 2025 Apr 23;641(8062):380–7. doi: 10.1038/s41586-025-08865-8 (PMC12058516; doi:10.1038/s41586-025-08865-8)
Supplement: Supplementary file 1 — Timepoint information for supplementary video files. [file 41586_2025_8865_MOESM1_ESM.docx]

Supplementary Information (SI) Guide

Supplemental Video1 – Inline animation of 3-D seismic volume.

Supplemental Video2 – Xline animation of 3-D seismic volume.

Supplemental Video3 – A 360° tour of the shape and geometry of the AML|LAB structures.

Supplemental Video4 – A 3-D tour highlighting areas of strong reflectivity beneath Axial volcano.

Supplemental Video5 – A 3-D tour of the shape and geometry of the AML|LAB structures beneath the southeast flank of Axial volcano.

----------------------------------------------------------------------------------------------------------------

Supplemental Video 1 Timepoint:

0 s **start** Inline slice 102

10 s Inline slice 201, domal shape of AML|LAB (Funnel A and B) beneath caldera becomes visible (center)

17 s Inline slice 275, centered on spine of Funnel B and melt ribbon (center-right)

19 s Inline slice 286, Fig. 2a, AML|LAB is 22.5 km in width; truncation of sills (left) becomes visible

22 s Inline slice 320, Funnel A disappears (left)

24 s Inline slice 355, Funnel B disappears (center-right), Funnel C appears (right)

28 s Inline slice 383, centered on spine of Funnel C AML|LAB beneath CoAxial Segment appears (far right)

34 s Inline slice 440, Funnel C disappears AML|LAB beneath CoAxial Segment appears (far right)

35 s Inline slice 450, dipping AML|LAB, east slope of north rift arm (left)

41 s Inline slice 515, appearance of AML|LAB at New Dymond (far right)

42 s Inline slice 525, appearance of CoAxial AML (center left)

43 s **end** Inline slice 535

Supplemental Video 2 Timepoint:

0 s **start** Xline slice 1,001

6 s Xline slice 1,621, Funnel D becomes visible (far right)

10 s Xline slice 1,747, Funnel C appears (center-right)

13 s Xline slice 1,828, Funnel D disappears (far right)

17 s Xline slice 1,954, bright sub-AML|LAB sill beneath Funnel C (center-right)

19 s Xline slice 2,010, perched AML-LAB Funnel C (center-right)

21 s Xline slice 2,060, Funnel B appears (center)

22 s Xline slice 2,070, Funnel C disappears (center-right)

23 s Xline slice 2,121, Funnel B at its widest, 5 km (center)

33 s Xline slice 2,424, Fig. 2b, AML|LAB is 4.2 km wide, truncated sills (center)

36 s Xline slice 2,521, Funnel B narrows forming melt ribbon (center)

43 s Xline slice 2,729, flat AML|LAB and sills underlying caldera (center)

47 s Xline slice 2,841, center of AML|LAB beneath caldera is depressed (travel time) (center)

53 s Xline slice 3,055, AML|LAB beneath CoAxial Segment appears (far right)

58 s Xline slice 3,171, AML|LAB beneath CoAxial Segment disappears (far right)

59 s Xline slice 3,191, Funnel A appears (center)

1:00 s Xline 3,251, AML|LAB beneath east flank of north rift arm appears (far right)

1:15 s Xline 3,626, AML|LAB beneath east flank of north rift arm disappears (far right)

1:07 s Xline slice 3,450, vertically stacked sills beneath Funnel A (center)

1:12 s Xline 3,556, Funnel A disappears (center)

1:15 s **end** Xline slice 4,200

Supplemental Video 3 Timepoint:

0 s **start** First 360° orbit around the 3-D volume (Funnels not visualised)

24 s Next orbit with AML|LAB Funnels A, B, C & D now visualised

29 s Best view of Funnel B (center), Funnel C (center-right) and Funnel D (far right)

38 s AML|LAB beneath eastern flank of north rift axis (center)

41 s Funnel A is now centered (front)

47 s Flat AML|LAB is now centered (front)

47 s **end** Best view of offset between Funnels B & C (center-right)

Supplemental Video 4 Timepoint:

0 s **start** reflection strength AML|LAB (Funnel B) southeast flank (center) and perched AML|LAB (Funnel C) seen (lower-right)

5 s side view of Funnel B looking northeast (center)

7 s side view of melt ribbon (center-left)

11 s view of reflection strength beneath caldera (center)

13 s view of reflection strength of Funnel A (center)

17 s **end**

Supplemental Video 5 Timepoint:

0 s **start** First partial orbit of southeast flank, Axial Volcano (center)

2 s best view of AML|LAB associated with Funnels B, C and D (center-right)

10 s side view of Funnel D (far left) and AML|LAB beneath east flank of north rift arm (far right)

11 s reverse direction, visualise Funnels B, C and D and AML|LAB east flank of north rift arm

16 s best view of Funnels B, C and D (center)

21 s **end** sideview of Funnels B, C and D (center to left)
